# Hologene 5: A Phase II/III Clinical Trial of Combined Cell and Gene Therapy of Junctional Epidermolysis Bullosa

**DOI:** 10.3389/fgene.2021.705019

**Published:** 2021-09-01

**Authors:** Laura De Rosa, Elena Enzo, Giulia Zardi, Christine Bodemer, Cristina Magnoni, Holm Schneider, Michele De Luca

**Affiliations:** ^1^Holostem Terapie Avanzate, s.r.l, Modena, Italy; ^2^Centre for Regenerative Medicine “Stefano Ferrari”, University of Modena and Reggio Emilia, Modena, Italy; ^3^Department of Statistical Sciences, University of Bologna, Bologna, Italy; ^4^Department of Dermatology, Necker Enfants Malades Hospital, APHP, University Paris Centre, ERN-Skin Network (European Network for Rare Skin Disorders), Paris, France; ^5^Unit of Dermatology, University of Modena and Reggio Emilia, Modena, Italy; ^6^Department of Pediatrics, University Hospital Erlangen, Erlangen, Germany

**Keywords:** epidermolysis bullosa, cell and gene therapy, epidermal stem cell, clinical trial, skin, genodermatoses

## Abstract

Epidermolysis bullosa (EB) is a group of devastating genetic diseases characterized by skin and mucosal fragility and formation of blisters, which develop either spontaneously or in response to minor mechanical trauma. There is no definitive therapy for any form of EB. Intermediate junctional EB (JEB) caused by mutations in the gene *LAMB3* has been the first genetic skin disease successfully tackled by *ex vivo* gene therapy. Here, we present a multicenter, open-label, uncontrolled phase II/III study that aims at confirming the efficacy of Hologene 5, a graft consisting of cultured transgenic keratinocytes and epidermal stem cells and meant to combine cell and gene therapy for the treatment of *LAMB3*-related JEB. Autologous clonogenic keratinocytes will be isolated from patients’ skin biopsies, genetically corrected with a gamma-retroviral vector (γRV) carrying the full-length human *LAMB3* cDNA and plated onto a fibrin support (144cm^2^). The transgenic epidermis will be transplanted onto surgically prepared selected skin areas of at least six JEB patients (four pediatric and two adults). Evaluation of clinical efficacy will include, as primary endpoint, a combination of clinical parameters, such as percentage of re-epithelialization, cellular, molecular, and functional parameters, mechanical stress tests, and patient-reported outcome (PRO), up to 12months after transplantation. Safety and further efficacy endpoints will also be assessed during the clinical trial and for additional 15years in an interventional non-pharmacological follow-up study. If successful, this clinical trial would provide a therapeutic option for skin lesions of JEB patients with *LAMB3* mutations and pave the way to a combined cell and gene therapy platform tackling other forms of EB and different genodermatoses.

Clinical Trial Registration: EudraCT Number: 2018-000261-36.

## Introduction

Epidermolysis bullosa (EB) is a heterogeneous group of rare, genetic disorders caused by molecular defects in genes encoding several structural proteins that form the epidermal-dermal junction. EB is characterized by mechanical fragility and recurrent blistering of the skin and other stratified epithelia (Bardhan et al., 2020).

Based on the level of skin cleavage, EB can be classified into four major types – EB simplex (EBS), junctional EB (JEB), dystrophic EB (DEB), and Kindler syndrome – that differ in severity and prevalence. Clinical manifestations range from mild to severe with local or generalized skin and mucosal involvement, depending on phenotypic and molecular factors (Fine, 2010; Fine et al., 2014; Has et al., 2020). The main clinical features of EB, such as blistering, erosions, and recurrent infections, can be associated with scarring, nail dystrophy, teeth abnormalities, milia, oral lesions, esophageal strictures, and thriving impairment. The severe scarring typical for recessively inherited DEB (RDEB) induces pseudosyndactyly, which contributes to the poor quality of life of RDEB patients. Patients suffering from intermediate JEB and RDEB are highly prone to develop aggressive squamous cell carcinomas (SCC) leading to premature death (Mallipeddi et al., 2004; Fine et al., 2009; Fine, 2010; Montaudie et al., 2016). Thus, depending upon the subtype, the prognosis of EB may range from a virtually normal life with only minor skin lesions to neonatal death due to severe skin and mucosal defects.

There is no cure for EB (Mellerio et al., 2020). Available therapies are largely palliative and aim to prevent blisters and/or promote their healing, but only partially alleviate the devastating clinical manifestations. Long-lasting, curative therapies are urgently needed and, nowadays, gene therapy in combination with stem cell-based strategies is still at the experimental stage.

## Rationale and Previous Clinical Studies

Recessively inherited JEB is caused by mutations in three genes, *LAMA3*, *LAMB3*, or *LAMC2*, that jointly encode laminin 332 (a heterotrimeric protein consisting of α3, β3, and γ2 chains, also known as laminin 5) and in genes encoding collagen XVII, α6β4, and α3 integrins. Laminin 332 mutations are usually associated with the most severe forms of JEB and mainly affect *LAMB3* (Fine et al., 2014; Has et al., 2020). Absence of laminin 332 due to deleterious (nonsense) mutations causes early lethality and defines severe JEB (also known as Herlitz form). In non-lethal intermediate JEB, laminin 332 is strongly reduced and hemidesmosomes are rudimentary or absent, which lead to recurrent blisters and chronic erosions greatly impairing patients’ quality of life (Kiritsi et al., 2013). More than 40% of patients suffering from intermediate JEB die before adolescence (Fine et al., 2008). The continuous inflammation, the disruption of the adhesive machinery and the proliferative pressure on epidermal cells induce the development of cancer in the wounded skin. Indeed, JEB patients have an increased risk (1:4) of developing SCC (Yuen and Jonkman, 2011; Montaudie et al., 2016). These tumors have a high recurrence rate and an aggressive course that results in premature death in approximately 20% of patients (Yuen and Jonkman, 2011).

Intermediate JEB caused by mutations in the gene *LAMB3* (*LAMB3*-JEB) was the first genetic skin disease successfully tackled by combined *ex vivo* cell and gene therapy. Autologous transgenic epidermal cultures were grafted on selected skin areas in two adult and one pediatric patients (Mavilio et al., 2006; Bauer et al., 2017; Hirsch et al., 2017; Table 1).

**Table 1:** Summary of clinical data on previously treated patients.

	1° Mavilio et al., 2006	2° Bauer et al., 2017	3° Hirsch et al., 2017
Type of application	Phase I/II	Single-case study	Compassionate use
Year of transplantation	2005	2014	2015
Type of mutation	Compound heterozygous: c.628G>A; c.29insC	Compound heterozygous: c.1903C>T; c.3009C>T	Homozygous: c.1977-1G>A
Total graft size	Ca. 500cm^2^ (0.05m^2^)	Ca. 80cm^2^ (0.008 m^2^)	0.85m^2^
Transplanted area	Right and left legs	Right leg	Arms, legs, flanks, back skin, and thigh
% of re-epithelization	100%	100%	100%
Admission	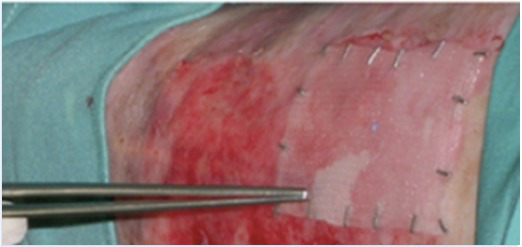	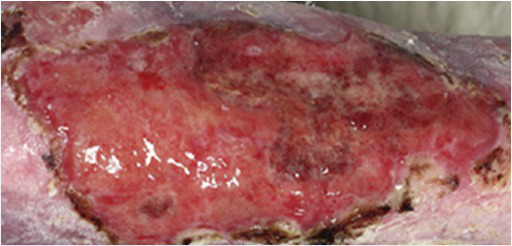	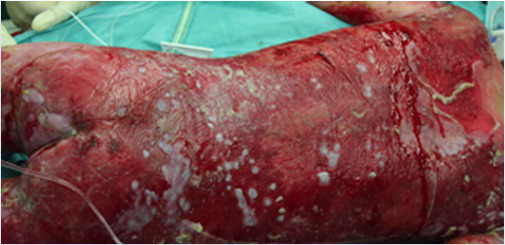
After treatment	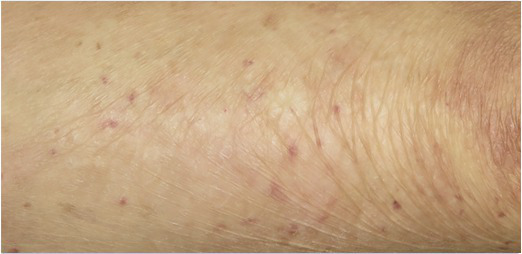	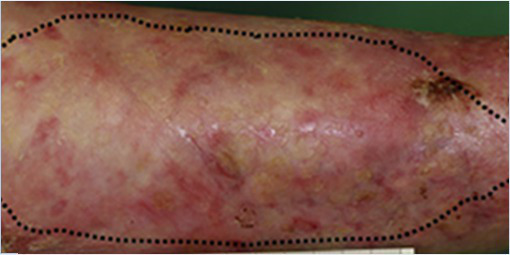	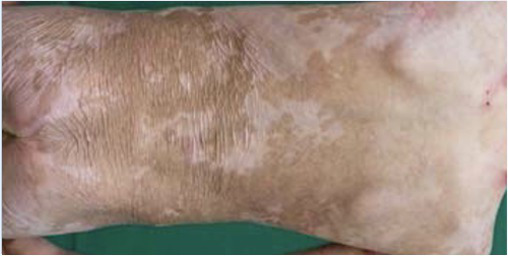
Presence of the transgene (mRNA and protein)	YES	YES	YES
Years of follow-up post treatment	16	2 (Patient not included in the observational study)	6

Figures from Mavilio et al., 2006 and Bauer et al., 2017 published with permission of Elsevier and Springer Nature.

The first two patients were treated using transgenic epidermal cells grown on plastic. Cohesive epidermal sheets were detached from the vessel and mounted on petrolatum gauze (Mavilio et al., 2006; Bauer et al., 2017). Patient 3 was also treated with grafts derived from the patient’s own epidermal stem cells corrected by retroviral gene transfer and with transgenic keratinocytes grown on a fibrin substrate (Hologene 5). Previous *in vitro* experiments and clinical application of plastic- and fibrin-cultured grafts on full-thickness skin burns have unambiguously shown that these culture systems are fully equivalent, both in terms of biological parameters (including preservation of epidermal stem cells) and clinical performance. Indeed, both plastic- and fibrin-cultured transgenic epidermal sheets were able to engraft and permanently and fully restore a functional epidermis (Pellegrini et al., 1999). Cultivation on fibrin has multiple advantages, including a better coordination between culture timing and surgery, an easier handling and long-distance transportation of the grafts, no time-consuming procedure for the detachment of the cultures, and, more importantly, it avoids the dispase-dependent shrinking of the epidermal sheet. The last feature allows preparation of 144cm^2^ grafts using the same number of clonogenic keratinocytes and the same amount of culture media needed to produce 50–60cm^2^ plastic-cultured grafts (Pellegrini et al., 1999). Thus, for this trial, we will use only Hologene 5.

Patient 1, a 36-year-old man, carries a null allele of *LAMB3* and a second allele with the *LAMB3* missense mutation E210K, which impaired normal assembly of laminin-332 (Mellerio et al., 1998; Posteraro et al., 2004). He showed blisters, arising spontaneously or upon minimal mechanical stress or trauma, on the majority of his body surface. In October 2005, the anterior upper parts of his legs were successfully treated with transgenic epidermal cultures (Mavilio et al., 2006; Table 1). A fully functional epidermis has been restored and no adverse events (AEs) were recorded during 6.5 years follow-up (De Rosa et al., 2014; Table 1). An ongoing observational study confirms both the stability of the transgenic epidermis and the absence of AEs 16years after transplantation. Patient 2, a 49-year-old woman, is a compound-heterozygous carrier of splice-site mutation (c.3009C>T) and a nonsense mutation (R635X) in *LAMB3* (Buchroithner et al., 2004). She had a large non-healing chronic wound on her lower right leg that was treated in June 2014. A functional epidermis could be restored also in this patient and no adverse events were reported after 2years follow-up (Bauer et al., 2017; Table 1).

Transgenic epidermal cultures have proven to be lifesaving in Patient 3, a 7-year-old boy carrying a homozygous acceptor splice site mutation (c.1977-1G>A) within intron 14 of *LAMB3* (Hirsch et al., 2017). Due to the severity of the disease and to infection with *Staphylococcus aureus* and *Pseudomonas aeruginosa* shortly after admission to hospital, he suffered complete epidermal loss on approximately 80% of his total body surface and the prognosis was very poor. Virtually his entire skin was replaced by approximately 1m^2^ of transgenic cultured epidermis, the majority of which consisted of Hologene 5 (Hirsch et al., 2017). At the last follow-up (5years after transplantation), the patient’s newly formed epidermis expressed normal levels of laminin 332, had an intact basement membrane, normal thickness, and morphology of hemidesmosomes, did not develop blisters or erosions, remained robust and resistant to mechanical stress and unveiled normal wound healing upon injury. The regenerated epidermis was entirely transgenic, as *LAMB3* mRNA and laminin 332 were uniformly and seamlessly detected in all the analyzed skin sections. No adverse events, immune response or inflammation were observed (Kueckelhaus et al. manuscript submitted).

Patients 1 and 3 are currently included in an observational study (HTA-HG5-01-OBS) started in July 2018 and still ongoing to collect further safety and efficacy data (Table 1).

These findings do not only attest a most likely permanent (16years so far) functional restoration of the epidermal-dermal junction but also reveal a promising safety profile. Indeed, despite the genotoxic risks linked to gamma-retroviral vector (γRV) insertional mutagenesis reported for hematopoietic stem cells (Wu and Dunbar, 2011), no clonal selection or immortalization events have been observed in epidermal stem cells. Altogether, the three JEB patients received ~4×10^8^ transgenic clonogenic keratinocytes, none of which provided evidence of clonal expansion, within a time frame that entailed over 150 epidermal renewal cycles (Mavilio et al., 2006; De Rosa et al., 2014; Bauer et al., 2017; Hirsch et al., 2017). Indeed, neither tumor development nor other adverse events have been observed in any of the treated areas. These data suggest that insertional mutagenesis is an extremely rare event (if present at all) in human epidermal cells. Most likely, to trigger tumor formation, insertional mutagenesis will require other oncogenic factors, which could be related to cell type, patient’ s individual genetic background, age, disease, transgene, or other mutations (Howe et al., 2008; Cavazza et al., 2013). In fact, we have never observed any immortalization or transformation event in cultured primary clonogenic keratinocytes despite hundreds of transduction procedures performed in 3 decades of basic research. Similarly, a group at Stanford University treated seven patients with epidermal cultures genetically corrected with a γRV carrying the *COL7A1* gene, and no adverse events were observed after 5 years of follow-up, irrespective of patients’ age (Siprashvili et al., 2010, 2016; Eichstadt et al., 2019).

Given the high likelihood of JEB and RDEB patients to develop aggressive SCC, often leading to their premature death, the rationale for performing this trial also in children – besides the obvious advantage of promptly restoring a functional epidermis, hence, preventing recurrent blisters, erosion, and chronic inflammation – arises from the likely assumption that gene therapy could actually decrease the risk of skin cancer development in the transplanted areas.

Despite these encouraging preliminary results, such experimental approaches are still far from a routine therapy. Therefore, the Hologene 5 trial aims to confirm the efficacy and to strengthen the safety of combined *ex vivo* cell and gene therapy of *LAMB3*-related JEB. If successful, this clinical trial would pave the way for the approval of Hologene 5 as a therapeutic option for at least one subgroup of patients with intermediate JEB.

## Materials and Methods

### Trial Design

In March 2015, Hologene 5 received an orphan designation (EU/3/15/1465) by the European Commission. This trial is a multicenter, open-label, uncontrolled phase II/III study designed to confirm efficacy and safety of Hologene 5 in patients with *LAMB3*-related JEB.

### Product Review and Drug Formulation

**Figure 1:**
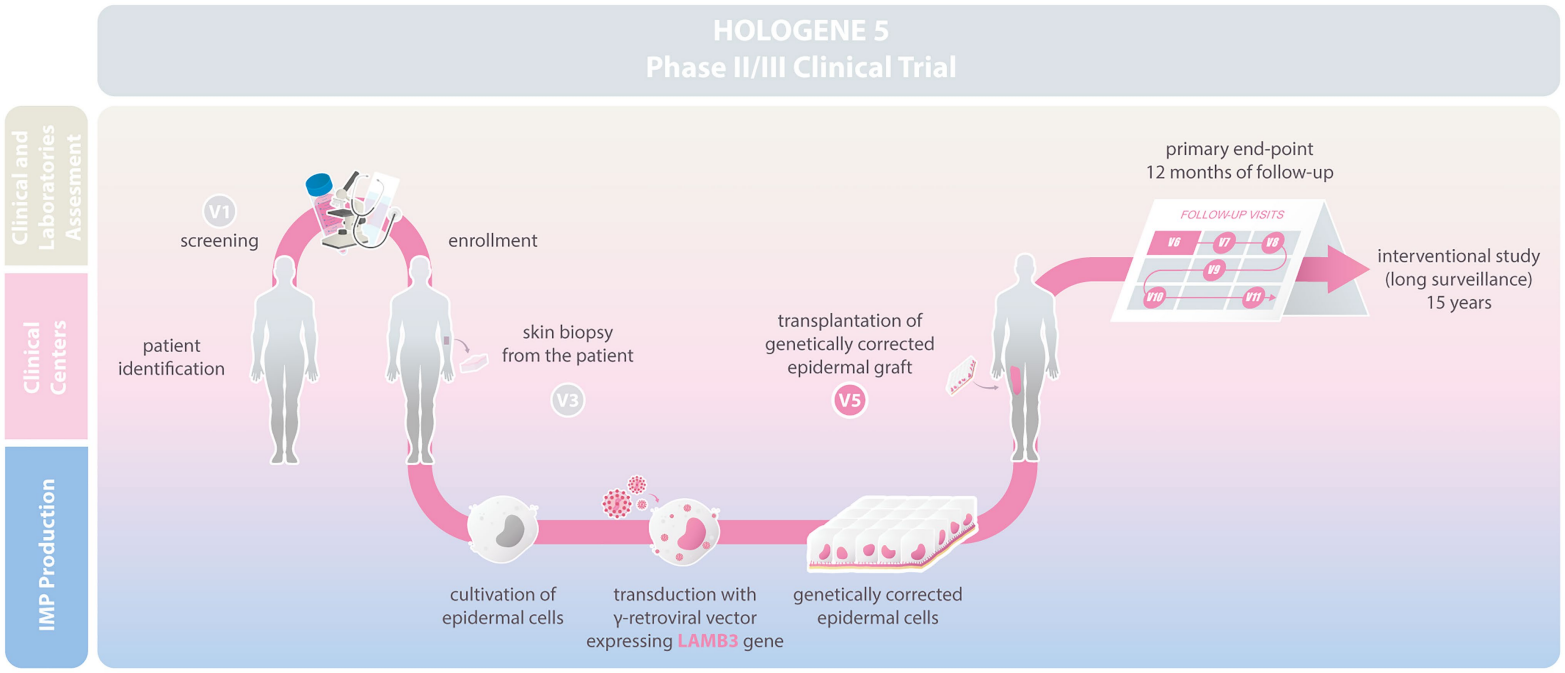
Scheme describing the Hologene 5 clinical trial. Junctional EB (JEB) patients will be enrolled at the clinical centers and skin biopsies will be sent to Holostem Terapie Avanzate s.r.l for Good Manufacturing Practice (GMP) manufacturing of Hologene 5. Clonogenic epidermal cells will be cultivated and genetically corrected with a gamma-retroviral vector (γRV) expressing LAMB3. Hologene 5 will be delivered to the clinical center for transplantation in V5. Safety and efficacy of Hologene 5 will be assessed for 12months based on a combined evaluation of clinical performance, molecular and functional parameters, and patient perception. Treated patients will be monitored for additional 15years in an interventional study.

Hologene 5 is an Investigational Medicinal Product (IMP; classified as Advanced Therapy Medicinal Product, ATMP), manufactured under Good Manufacturing Practice (GMP) conditions by Holostem Terapie Avanzate s.r.l. as a sterile, 144cm^2^ epidermal graft. It consists of fibrin-cultured epidermal keratinocytes transduced with a γRV carrying a *LAMB3* cDNA (Figure 1). Autologous primary clonogenic keratinocytes will first be expanded *ex vivo* and genetically corrected with the *LAMB3*-γRV (Drug Substance). Hologene 5 will then be prepared by plating 50.000–150.000 transduced cells/cm^2^ on a 144cm^2^ fibrin support to generate transplantable grafts, each containing 20–30.000.000 viable transgenic keratinocytes (Drug Product; Hirsch et al., 2017; Figure 1). Such grafts must contain an adequate number of transduced epidermal stem cells, which are instrumental to achieve a long-lasting skin regeneration and proper wound healing.

Hologene 5 will be delivered to the clinical center using a temperature controlled proprietary packaging system that preserves the graft up to 36h after packaging. Hologene 5 is intended for transplantation onto surgically prepared blistering skin areas of JEB patients to obtain a permanent regeneration of a healthy, functional, and renewing epidermis. Each patient will be treated with one or more grafts according to wound size and number, and each transplanted area will be considered as a single treatment regardless the number of grafts used (see below).

### Recruitment, Trial Population, and Eligibility Criteria

This trial is designed to enroll at least six *LAMB3*-JEB patients (four pediatric and two adult). Their enrollment will occur at study sites, where the respective patients have already been registered and cared for. Each center will identify and pre-screen suitable patients taking into account their clinical history. The selection will be carried out according to stringent inclusion/exclusion criteria, as summarized in Table 2. If the criteria are met, patients of both genders and of any ethnic origin will be considered for the study. Selected patients will receive a personal invitation from the local investigators and will only be included after written informed consent. They will be followed at the study site where they were enrolled. Patients can withdraw from the study at any time.

**Table 2:** Eligibility criteria for Hologene 5.

**Inclusion criteria**	• Male and female patients between 6months and 65years old
	• Diagnosis of intermediate *LAMB3*-related JEB, confirmed by DNA sequencing and/or immunofluorescence analysis of skin biopsy sample(s)
	• Presence of blisters and/or erosions ≥6 cm^2^, persistent or recurrent for more than 3months
	• Cooperative attitude and patient's compliance with scheduled visits
	• Detectable residual expression of laminin-332 and its beta-3 chain by immunofluorescence and/or Western blot analysis
**Exclusion criteria**	• Known or suspected intolerance to anaesthesia or to study medication excipients or other material required by study protocol
	• Bad general condition (ECOG index >1) and/or acute systemic infection at the time of screening (patient can be re-screened after appropriate treatment)
	• Presence of any skin cancers in the area(s) qualifying for treatment
	• Female subject who is pregnant
	• Presence of (i) systemic diseases, (ii) clinically significant or unstable concurrent disease, (iii) other concomitant medical conditions, and (iv) other clinical contraindications to stem cell transplantation, based on investigator’s judgment, and consultation with the sponsor's medical expert
	• Previous treatments or clinical trials envisaging the use of cells (including bone marrow transplantation) and/or *in vivo* or *ex vivo* gene therapy products

### Study Treatment

Skin areas suitable for transplantation will be identified and evaluated during the screening visit and monitored at each study visit. The selection of such areas shall be based on specific parameters like severity (areas with open wounds and blistering should be chosen; see inclusion criteria), impact on quality of life (for instance pain and recurrent infections), accessibility for local anesthesia, ease of immobilization, and management after grafting. The patient’s opinion will also be taken into account.

The area(s) to be treated by transplantation, as well as the control area to be used for intra-patient treatment comparison (exploratory endpoint), will be confirmed at **Visit 5** (see below). If multiple control areas are used and the clinical performance of the treated areas is satisfactory, some of the control areas can be considered for Hologene 5 transplantation, even before the end of the trial.

Hologene 5 will be administered by qualified surgeons under standard sterile operating conditions. Transplantation can be done under local or general anesthesia. To remove remnants of affected epidermis, Erbium YAG laser or copper filament sponge or surgical knife can be used. Hologene 5 will be applied on the prepared wound bed; the grafts will be covered with petrolatum gauze overlaid by a firm but non-compressive standard bandage to ensure proper engraftment and fibrin absorption. After treatment, patients should preferably remain hospitalized and the treated areas are immobilized up to 2weeks (if deemed necessary). The immobilization of the treated body part is required to ensure proper engraftment. Control skin areas will be treated with standard medications and bandages.

### Primary, Secondary, and Exploratory Objectives

The primary objective of the clinical trial is to evaluate the efficacy of Hologene 5, defined as proportion of success based on clinical performance, cellular, molecular, and functional parameters, and patient-reported outcome (PRO).

Secondary objectives are the efficacy and the safety of Hologene 5 according to combined visual and microscopic analysis of the re-epithelialized area, measured both by the Investigator and an Independent Assessor.

The exploratory objectives are the assessment of safety and efficacy of Hologene 5 based on intra-patient’s comparison and the percentage of patients with successfully treated areas, absence of immune response against the therapy and amelioration of patients’ quality of life.

### Primary Endpoint

**Figure 2:**
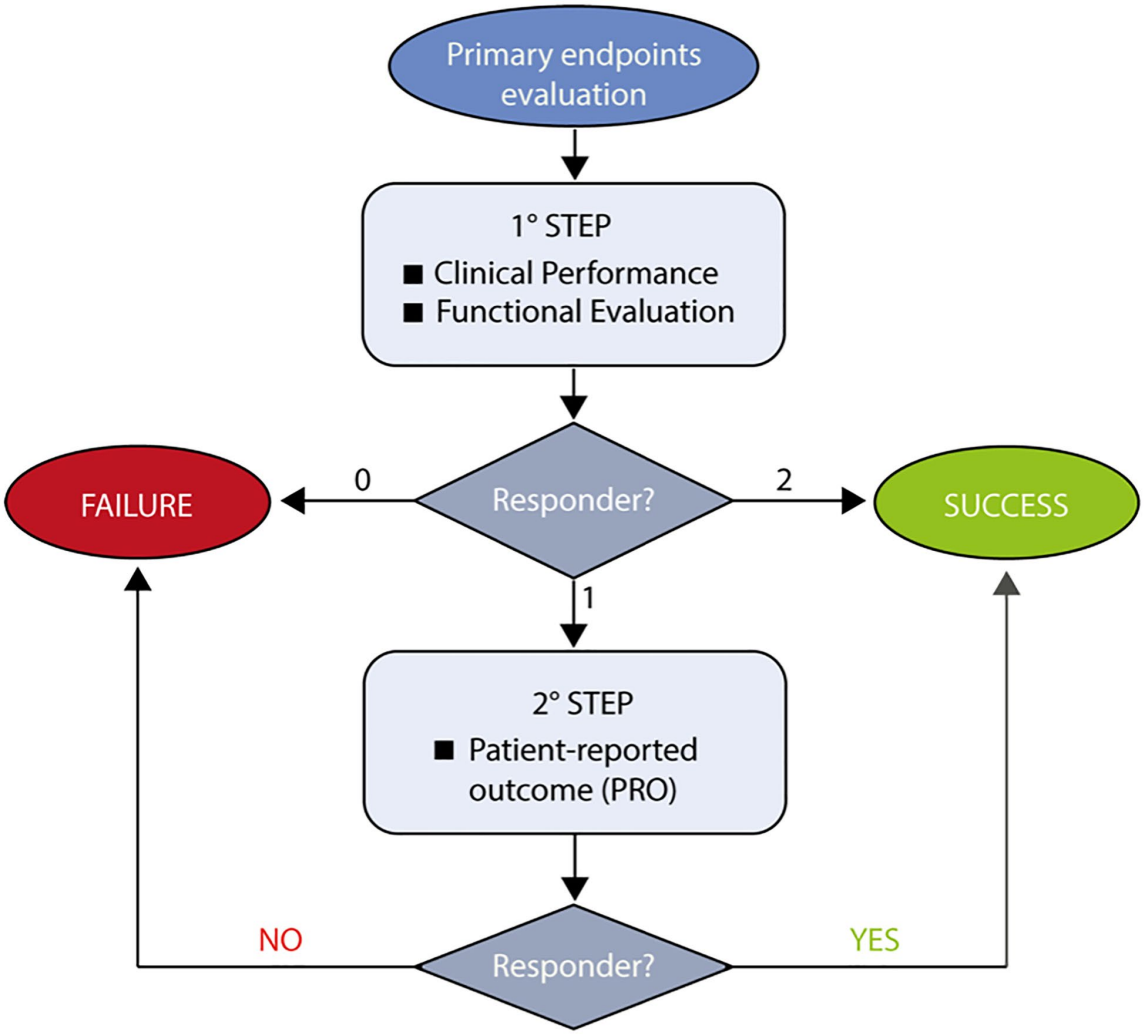
Flowchart describing assessment of Hologene 5 efficacy. The evaluation of Hologene 5 efficacy is the primary endpoint of this clinical trial and will follow a two-step rule. At the first step, clinical (percentage of re-epithelialization) and functional (presence of a transgenic functional epidermis) parameters will be evaluated. If both criteria are met, the treatment will be defined as Success; if none of them is fulfilled, the treatment will be scored as Failure. In case only one criterion is met, a patient-reported outcome (PRO) will be considered (2 step). If PRO is positive, the treatment can still be defined as Success.

The primary endpoint is the proportion of transplantation success after 12months of treatment, defined through a two-steps rule (Figure 2).

An appropriate assessment of the efficacy of Hologene 5 cannot simply rely on re-epithelialization (as stand-alone primary endpoint), since JEB skin lesions can heal spontaneously, though temporarily. Thus, in order to rigorously evaluate Hologene 5 efficacy, the primary endpoint (Figure 2) will take into account not only clinical parameters (as the percentage of re-epithelialization measured by the Investigator) but also cellular, molecular, and functional parameters (by means of *in situ* hybridization, PCR, immunofluorescence and transmission electron microscopy) to verify the presence of *LAMB3* mRNA, integrated transgene, laminin 332 and β3 proteins and mature hemidesmosomes, mechanical stress assays (as stripping tests), and PRO [Likert scale to provide their perception of treatment success on the transplanted area(s)].

### Secondary Endpoints

The percentage of re-epithelialization will be assessed through clinical inspection and computer-assisted image analysis (iMitoWound digital imaging software), at 1, 3, 6, 9, and 12months by the Investigator and a trained Independent Assessor.

The absence of sub-blisters in the treated areas will be evaluated by Optical Coherence Tomography (OCT) at the end of the study. Changes in quality of treated area(s), from baseline to 12months follow-up, will be analyzed according to the Epidermolysis Bullosa Disease Activity and Scarring Index (EBDASI) validated questionnaire (Loh et al., 2014).

The incidence of adverse events (TEAEs), serious adverse events (SAEs), event of special interest (AESI), and adverse drug reactions (ADRs), vital signs and laboratory parameters will also be analyzed as secondary safety endpoints.

### Exploratory Endpoints

As exploratory endpoints, patients will be monitored for the presence of any immune response (B- or T-cell-mediated) against the transgene. Change in patients quality of life will also be evaluated through a specific questionnaire, Quality of Life Evaluation in Epidermolysis Bullosa (QOLEB; Frew et al., 2009), in case of multiple treatments involving a large body area. Finally, an intra-patient comparison will be performed between treated and non-treated areas and the percentage of patients with one or more successful transplantation(s) among all treated patients will be determined.

### Study Protocol Outline

At least six patients, four pediatric (6month- to 17-year-old), and two adults will be enrolled in the study. During the screening phase (**Visit 1–2**), the subjects will be examined at the centers involved to confirm the clinical diagnosis of intermediate JEB. They will be properly and fully informed and invited to sign the informed consent/assent (ICF/IAF). For children only, parents shall act as deputy. In the absence of a molecular diagnosis, a blood sample will be taken to determine the mutation through NGS analysis and confirm the involvement of the *LAMB3* gene. Two skin punch biopsies will be taken in order to verify the residual expression of laminin 332 and β3 chain by both immunofluorescence on skin sections and Western blot on primary keratinocytes cultures (Table 2, inclusion criteria). All clinical and molecular data previously collected from the patients will be analyzed (**Visit 1–2**). Whenever possible, previous testing that is documented and satisfies screening requirements will be considered, in order to limit the number of skin biopsies and the quantity of blood required from the patient.

**Figure 3:**
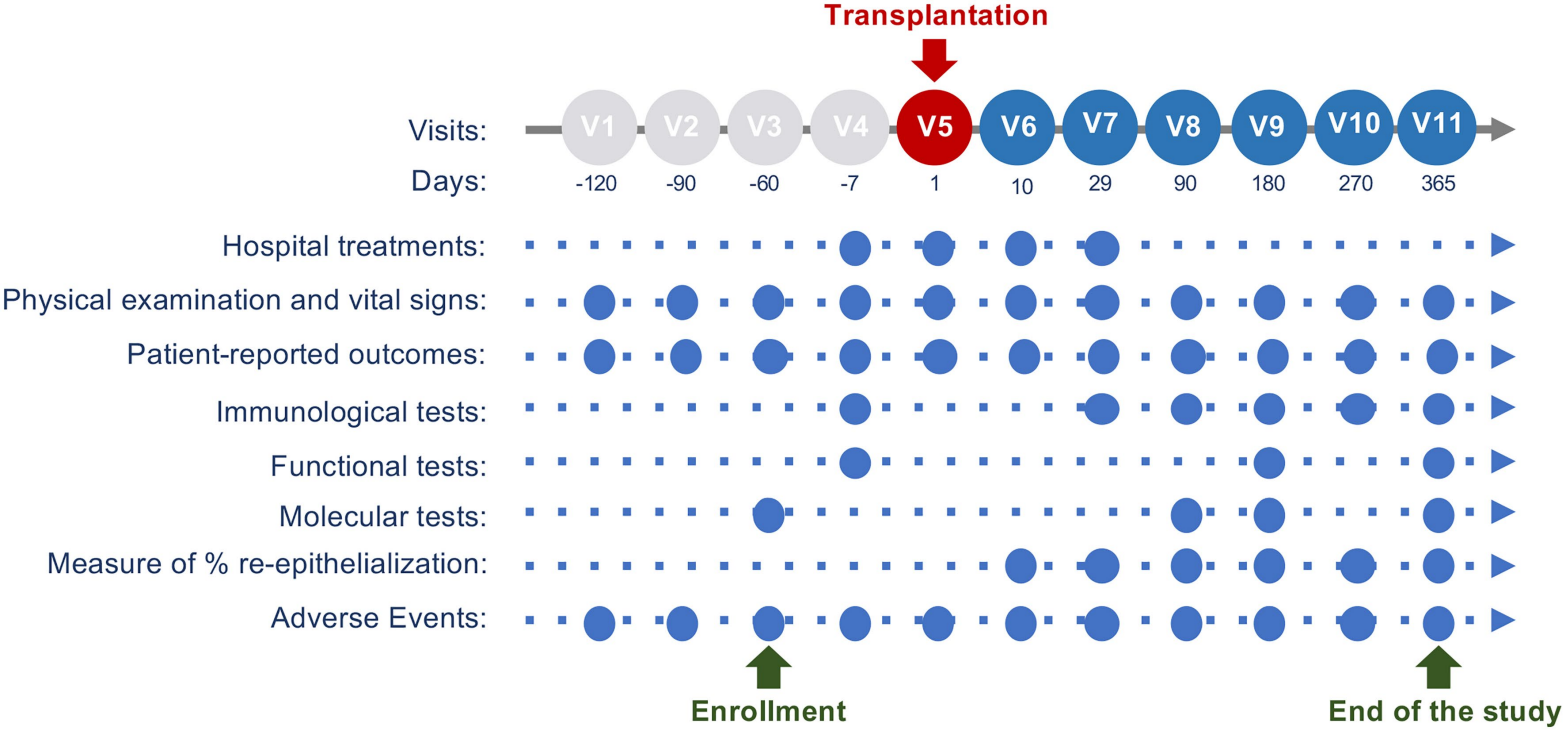
Study visit schedule. This scheme describes the main activities performed at each visit. Pre-treatment phase is from approximately 6months (V1) to approximately 7days before the transplantation (V4). Hologene 5 transplantation will be performed during V5 (Day 1). Follow-up visits are planned from approximately 10days after the grafting (V6) to the endpoint, 12months after the transplantation (V11).

At **Visit 1**, the complete medical and surgical history of the patient is recorded. Skin physical examination will be performed and digital skin photographs will be collected using digital imaging software (iMitoWound; Figure 3). All areas suitable for transplantation will be scored using EBDASI (**Visit 1** to **Visit 4**). After ICF/IAF signature, AE will be registered and assessed through the study (up to **Visit 11**) as well as any changes in concomitant medications.

A self-monitoring disease assessment has been introduced in this study in order to provide a fast track record and report every adverse event. The patient is provided with a tablet containing a user-friendly App (HoloApp, developed by the Sponsor) as patients’ diary for self-monitoring of their skin. HoloApp is designed for remote collection of digital images of the area(s) before and after transplantation. HoloApp will help patients to record any changes in the concomitant medications and wounds management and any adverse event to foster a quick Investigator’s action, if needed. Pictures of the transplanted and control area(s) are directly collected in a structured and pseudo-anonymized archive. HoloApp is not aimed to provide any diagnosis or to evaluate the Quality of Life.

At **Visit 3** (Day −60±30 before transplantation, Figure 3), one or two skin biopsy sample(s) of 2–9cm^2^ will be taken from non-blistering areas and sent to Holostem Terapie Avanzate s.r.l for Hologene 5 manufacturing. If needed, one or two (depending on the quality of the donor’s epidermal tissue) additional punch biopsies will be performed on the affected area for transmission electron microscopy, *in situ* hybridization and/or PCR analysis.

At **Visit 4** (3–7days before transplantation), the patient will be hospitalized and prepared for the surgical procedure. Areas to be treated by transplantation will be analyzed through iMitoWound and smear/swabs for bacterial cultures will be taken from the selected areas as well as from axilla, groin, and nose. In case of any positivity, based on Investigator’s judgment, a specific antibiotic treatment will be initiated. QOLEB will be administered to patients undergoing transplantation on large body areas (or candidates for multiple transplantations) and its outcome will be assessed 12months after the last transplantation.

At **Visit 5**, Hologene 5 transplantation will be performed (Day 1). Physical examination [e.g., Eastern Cooperative Oncology Group (ECOG) index] and vital signs collection (SBP, DBP, PR, and temperature) will be performed. A collection of digital images of control and areas to be treated will be taken through iMitoWound. These images will be considered as the reference baseline for the calculation of the percentage of re-epithelialization from **Visit 6** onward. Clinical staff in the operating room will take the pictures before and after preparation of the wound bed. The patient’s immune profile (presence of IgM and/or IgG against laminin 332 and T cell profile) will be assessed. A peripheral blood sample and one punch biopsy from peri-lesional skin wound areas selected for transplantation will be obtained and specific tests will be performed to monitor the immune response (see below).

Hologene 5 will be transplanted by qualified surgeons under standard sterile operating condition on the surgically prepared wound bed. Transplantation can be done under local or general anesthesia. Smear/swabs for bacterial cultures from all areas to be treated by transplantation will be taken to ensure, in the case of positivity, proper post-treatment antibiotic medication. At each administration, one or more Hologene 5 graft will be transplanted. Each graft will be adapted to the size of the skin lesion by removing the remnants using surgery scissors. The treated areas will be immobilized and covered with Adaptic petrolatum gauze overlaid by non-compressive standard bandage.

Based on Investigator’s judgment, in consultation with the Sponsor Medical Expert, patients may receive (if needed) post-transplantation medications, as corticosteroids, immune-suppressants, and/or immune-modulators. In fact, the first three patients treated with transgenic epidermal cultures (see previous clinical data) did not require such medications.

At **Visit 6** (8–10days after grafting), Hologene 5 engraftment will be evaluated both through physical examination and iMitoWound analysis. A blood sample will be collected to monitor potential immune reactions (presence of IgM and/or IgG to Laminin 332). The EBDASI questionnaire will be administered at each visit, from **Visit 6** to **Visit 11**.

At **Visit 7** (approximately 30days after transplantation), the Investigator will evaluate the percentage of re-epithelialization and the presence of blisters and erosions through physical examination and iMitoWound analysis, both in treated and control area(s). In case of any adverse reaction (e.g., inflammation, turgor, or any other suspicious signs), a punch biopsy and a blood sample may be taken for immunological assessment (Figure 3).

At **Visit 8** (approximately 90days after transplantation), the Investigator will evaluate the re-epithelialization of both treated and control area(s) through physical examination and iMitoWound analysis. To confirm and validate Hologene 5 efficacy, punch biopsies of the treated area will be used to investigate the expression of the transgene and the proper assembly of laminin 332. *In situ* hybridization and/or PCR analysis will be performed using a transgene-specific probe and immunofluorescence investigations will be conducted on 7-μm skin sections using both β3- and laminin 332-specific antibodies. A blood sample will be collected for immunological tests (presence of IgM and/or IgG against laminin 332).

At **Visit 9** (day 180) and **Visit 10** (day 270), the patient will be clinically monitored through physical examination and iMitoWound analysis. To verify the mechanical strength of the regenerated skin, a stripping test will be performed, but only in case of evident clinical success of the treatment. Based on Investigator’s judgment, in consultation with the Sponsor Medical Expert, additional punch biopsy samples can be taken to confirm the expression of the transgene and the proper assembly of laminin 332 by *in situ* hybridization and/or PCR and immunofluorescence. Such biopsies will be avoided in case of evident failure or if they are considered unnecessary.

The study will end at **Visit 11**, 12months after the transplantation (Figure 3). At this time point, the Investigator will evaluate the final clinical efficacy through physical examination and iMitoWound analysis, both in treated and control area(s). The skin architecture will be investigated through OCT, which allows the detection of epidermal sub-blisters or microscopic blisters that are not identifiable by visual inspection. The regeneration of a transgenic epidermis will be confirmed at a molecular level by *in situ* hybridization and/or PCR (using a transgene-specific probe) and immunofluorescence (using β3- and laminin 332-specific antibodies) performed on punch biopsies. In addition, the proper assembly of a correct number of mature hemidesmosomes will be evaluated by transmission electron microscopy. Finally, mechanical properties of the regenerated transgenic epidermis will be assessed by a stripping test assay. A PRO will be collected using a 1–5 Likert scale based on patient’s self-perceived level of improvement of the treated area(s) and the QOLEB will be administered to patients who underwent transplantation on large body areas or multiple grafting procedures.

Punch biopsies on transplanted areas will be avoided in case of evident failure of the treatment or if considered unnecessary. If needed, additional punch biopsies, as well as blood samples, can be taken and used to assess a potential immune reaction (presence of IgM and/or IgG against laminin 332).

### Safety and Laboratory Assessments

As indicated at each Visit, a set of morphological, functional, and molecular tests will be performed to evaluate the safety and efficacy of Hologene 5. Standard Operating Procedures are set in the specialized laboratories involved in the study, in agreement with current Good Laboratory Practices rules.

Immunofluorescence, *in situ* hybridization and PCR analyses will take place at the Centre for Regenerative Medicine (CMR) at the University of Modena and Reggio Emilia. The absence of replication-competent retrovirus (RCR) will be confirmed by qPCR on blood samples at CMR. Transmission Electron Microscopy will be performed at the laboratory of Biology and Pathology of soft Connective Tissues (BioPaCT) at the same University.

Immunological tests to monitor both immune response and specific T-cell activation against β3 chain and/or laminin 332 will take place at the MiTiCi laboratory, San Raffaele Hospital in Milan (Italy). Blood samples will be collected both pre- and post-transplantation for standard blood tests and detection of infectious agents (including SARS-CoV-2). The volume of blood samples will be compliant with European Ethical rules, in particular for pediatric patients.

To evaluate the B cell response, indirect immunofluorescence (IIF), and ELISA will be used to test for presence of autoantibodies against laminin 332 (Abs-LM332) in the patient’s plasma. Patient-specific baseline reactivity will be defined from plasma samples taken before grafting (**Visit 4**) and compared with samples collected after transplantation, from **Visit 6** to **Visit 11**. To investigate a potential local immune response, direct immunofluorescence (DIF) for IgM or IgG detection will be performed on skin sections derived from the selected areas, before and after transplantation. To evaluate patients’ β3-specific T-cell reactivity, a set of cytokines, including IFN-γ, IL-4/IL-13, and IL-17, and CD107a will be investigated before and after transplantation through ELISpot and flow cytometry assays. Percentage of CD4+ T regulatory and CD8+ T follicular helper cells will be also assessed. A minimum of 10–15ml of blood (three aliquots) is required from pediatric patients (based on age) and 30ml (six aliquots) from adult patients. Minors below the age of 2years are excluded from this test.

## Statistics

### Data Storage

All parties will ensure the protection of patients’ personal data and identifiable personal data will not be included in any report, publication, or other disclosure, except when required by law. Data will be securely stored, both on paper and on password-protected computers, with locked doors and shelves, accessible only to dedicated personnel. After study completion, all documents and study data will be kept by the Investigators in a secure and ordered study file for a minimum of 30years. It is the responsibility of the Sponsor to inform the Investigators on timing and modalities of data storage. The Investigators will contact the Sponsor before destroying any trial-related documentation.

### Sample Size Calculation

This study is designed to enroll at least six patients, with no formal estimation of sample size performed. The total number of six patients was defined based on the incidence of *LAMB3*-dependent intermediate JEB in Europe as indicated by Orphanet (Fine, 2010; Bardhan et al., 2020).

Given the exploratory nature and the characteristics of this study, rather than testing a formal hypothesis, a justification based on epidemiologic data was considered more appropriate than a power calculation in the traditional fashion or using an estimation approach through the precision of confidence intervals. The target population is a subgroup of the JEB, referred to as intermediate JEB. Intermediate JEB affects about 0.1 in 1 million people. Approximately 80% of such patients have a mutation in *LAMB3*, the gene encoding the β3 chain of laminin 332 (Kiritsi et al., 2013). This makes the estimate to be around 0.06–0.08 patients per million, i.e., no more than 40 patients in the total EU population (data at 1-01-2019; EUROSTAT, 2019). Intermediate JEB patients show an increased risk of developing SCC, especially in adulthood (Fine et al., 2008; Yuen and Jonkman, 2011; Fine, 2012). Since the presence of SCC in the areas chosen for transplantation is an exclusion criterion and only patients expressing detectable amounts of laminin 5 are enrolled, the total number of six patients (four pediatric and two adults) can be considered as a realistic estimate of the eligible patients, although this number is exceptionally small.

### Planned Statistical Analyses

General descriptive statistics for continuous variables will include number of observations (n), mean, SD, median, interquartile range (IQR), minimum and maximum values. Categorical data will be summarized by frequency and percentages.

The primary endpoint as the proportion of transplantation success (according to the two-steps rule) at 12months will be presented together with the two-sided 95% CI. The analysis of the proportion of transplantation successes will be performed in the Full Analysis Set (FAS; as primary analysis) at transplantation level, that is, all transplantations of all patients who undergo at least one application of the investigational product and in the Per Protocol Set (as sensitivity analysis) at transplantation level.

The proportion of patients with one or more transplantation success (according to the two-steps rule) will be presented together with the two-sided 95% CI. This analysis will be performed on the FAS set at patient level. In addition to descriptive statistics, when applicable, appropriate non-parametric statistical tests will be provided for secondary efficacy variables.

An overview of AEs with the number of patients affected, as well as the number of events, with any treatment emergent events (TEAEs), serious TEAEs, drug-related TEAEs, serious drug-related TEAEs, TEAEs leading to withdrawal, and TEAEs leading to death will be presented.

Treatment emergent events will be tabulated by system organ class (SOC) and preferred term (PT) using the MedDRA dictionary. Vital signs (actual values and change from baseline, if applicable) will be presented by time point. Safety laboratory data (actual values and change from baseline, if applicable) will be presented by time point.

A detailed statistical analysis plan will be finalized before database locking.

## Trial Management

### Governance and Monitoring

Holostem Terapie Avanzate s.r.l. is responsible for the governance of the trial. A monitoring plan has been established. All monitoring will be performed by an independent contract research organization (CRO). The CRO will contact the investigator/center before and during the study and survey the study records (including eCFR, study file, and source data) to monitor the progress and the management of the study and to address and discuss any emergent problem.

### Good Practices

Hologene 5 will be manufactured by Holostem Terapie Avanzate following full current GMP rules. All study procedures will adhere to Good Clinical Practices (GCP) and Good Clinical Laboratory Practice (GCLP) according to manufacturing or laboratory-specific standards. All legal and regulatory requirements will be accomplished.

### Potential Pitfalls

In patients with JEB, skin wounds are more likely to become infected than in otherwise healthy individuals, and entire epithelial grafts may get lost due to secondary infection despite successful transplantation. This might have impact on the primary endpoint of the trial. Therefore, rigorous systemic antibiotic treatment of any documented or suspected wound infection will be required.

### Ethics and Dissemination

The study will be conducted in agreement with the Declaration of Helsinki for patient care. Before beginning the trial, all ethical and regulatory approvals will be obtained. Results of the study, both negative and positive, will be published and/or presented at scientific meetings after the end of the study.

### Registration and Trial Status

The clinical study protocol is identified by the EUDRACT number 2018-000261-36 and has been included in the EU Clinical Trial Registry. Additional registration in the ClinicalTrials.gov database has also been initiated.

## Conclusion

Hologene 5 is an ATMP consisting of fibrin-cultured epidermal sheets generated by transgenic clonogenic keratinocytes, including epidermal stem cells. Given the complexity of the technology, the clinical features of JEB and the quite high cost of ATMPs, the cumbersome development of a product like Hologene 5 becomes acceptable if a full and permanent restoration of a functional epidermis is achieved (Mavilio et al., 2006; De Rosa et al., 2014; Bauer et al., 2017; Hirsch et al., 2017). Although, previous studies have shown that this can indeed be the case, we will be very stringent in determining the criteria for claiming efficacy of Hologene 5 and use a two-steps rule based on combined evaluation of primary endpoints, such as clinical performance, molecular and functional parameters, and patient perception. In fact, because of the intrinsic nature of the pathology, re-epithelialization of the treated areas, though being a necessary and crucial parameter, *per se* is not sufficient to score a positive clinical outcome, hence to claim efficacy of treatment. Nowadays, JEB (and RDEB) patients rely on many sophisticated supportive medications fostering a satisfactory, yet only temporary, wound closure (especially when patients are hospitalized) leading to continuous rounds of blistering and healing (Mellerio et al., 1998; Goldschneider et al., 2014; Bruckner-Tuderman, 2019). Hence, the simple measurement of the percentage of re-epithelialization in treated areas at a given moment could lead to a misleading positive score of Hologene 5 efficacy.

In order for Hologene 5 to be judged as efficacious, the newly formed epidermis should (i) be entirely transgenic, (ii) produce physiologic amounts of laminin 332 properly and seamlessly located within the basement membrane at the epidermal-dermal junction, (iii) restore a normal number of mature and functional hemidesmosomes, (iv) be robust, non-blistering, fully resistant to mechanical stress and able to heal wounds, and (v) be long-lasting (ideally for the lifetime of the patient), hence, contain engrafted transgenic epidermal stem cells allowing continuous self-renewal. In the three JEB patients treated so far, we have used these criteria for the evaluation of efficacy (Mavilio et al., 2006; De Rosa et al., 2014; Bauer et al., 2017; Hirsch et al., 2017; unpublished own data).

The remarkable clinical outcomes of regenerative medicine in renewing tissues, such as blood and squamous epithelia, accrue from a thorough characterization of their specific stem cells (De Luca et al., 2019; De Rosa et al., 2020). Thus, targeting stem cells is the cornerstone for successful *ex vivo* gene therapy of genetic diseases affecting the epidermis. Previous clinical data generated using Hologene 5 in *LAMB3*-JEB patients have indeed shown that the long-term stability of a fully functional transgenic epidermis is solely sustained by a defined population of stem cells, which have been identified as holoclone-forming cells, represent only a small percentage (approximately 5%) of all clonogenic keratinocytes and continuously generate pools of short-lived progenitors eventually producing terminally differentiated suprabasal cells (Hirsch et al., 2017). Thus, transgenic holoclone-forming cells must be contained in Hologene 5 as well in any other transgenic epidermal culture aimed at *ex vivo* gene therapy of any form of EB.

Keratinocyte cultures have long been safely used worldwide for life-saving permanent regeneration of a functional epidermis in massive full-thickness skin burns (Gallico et al., 1984; Pellegrini et al., 1999; De Luca et al., 2006) and for corneal restoration in ocular chemical burns associated to limbal stem cell deficiency (Pellegrini et al., 1997, 2013). Serious adverse events have never been reported. The transplanted cultured epidermis can be easily monitored throughout the lifetime of the patients and removed anytime, should adverse events occur. Accordingly, previous clinical studies using transgenic epidermal cultures have shown a good safety profile for both JEB and RDEB *ex vivo* gene therapy (Mavilio et al., 2006; De Rosa et al., 2014; Siprashvili et al., 2016; Bauer et al., 2017; Hirsch et al., 2017; Eichstadt et al., 2019). No adverse events have been reported, particularly no insertional mutagenesis (see Rationale for the clinical trial) and immune reaction against the cultured cells. Nevertheless, we will include in this trial only patients carrying *LAMB3* mutations that allow a residual, though minimal, expression of laminin 332. A potential immune reaction to Hologene 5 will be anyhow analyzed. In fact, the outcome of these studies might pose the basis for a potential use of Hologene 5 also in some selected severe forms of JEB, which are characterized by undetectable expression of laminin 332 (Kopp et al., 2005; Hammersen et al., 2016). In this respect, it has been shown that gentamicin can induce read-through of nonsense mutations and partially restore the expression of laminin 332 in patients with double null mutations in *LAMB3* alleles, without triggering any immune reaction (Hammersen et al., 2019; Kwong et al., 2020).

Hologene 5 represents probably the most advanced gene therapy approach for JEB. Hologene 5 is a complex product that combines cell therapy, gene transfer, and tissue engineering. This pivotal phase II/III clinical trial aims to strengthen its safety and confirm its efficacy, to obtain a formal approval of its use in patients affected by intermediate *LAMB3*-JEB. The knowledge and experience acquired during the development of Hologene 5 is currently driving several clinical trials tackling other forms of epidermolysis bullosa.

## Author Contributions

LDR and MDL designed the study protocol. CB, CM, and HS revised the study protocol. LDR and EE wrote the first draft of the manuscript. GZ planned the statistical analysis. All authors contributed to the article and approved the submitted version.

## Conflict of Interest

MDL is co-founder and member of the Board of Directors of Holostem Terapie Avanzate (HTA), s.r.l, Modena, Italy, as well as consultants for J-TEC-Japan Tissue Engineering, Ltd. LDR is HTA employee since 2018.

The remaining authors declare that the research was conducted in the absence of any commercial or financial relationships that could be construed as a potential conflict of interest.

## Publisher’s Note

All claims expressed in this article are solely those of the authors and do not necessarily represent those of their affiliated organizations, or those of the publisher, the editors and the reviewers. Any product that may be evaluated in this article, or claim that may be made by its manufacturer, is not guaranteed or endorsed by the publisher.
